# A Prospective Randomized Comparative Study Between Midvastus and Standard Medial Parapatellar Approaches for Total Knee Replacement Regarding the Peri-Operative Factors

**DOI:** 10.7759/cureus.29889

**Published:** 2022-10-03

**Authors:** Angelo V Vasiliadis, Vasiliki Chatziravdeli, Dimitrios Metaxiotis, Anastasios Beletsiotis

**Affiliations:** 1 2nd Orthopaedic Department, General Hospital of Thessaloniki “Papageorgiou”, Thessaloniki, GRC; 2 Orthopaedic Surgery and Sports Medicine Department, Croix-Rousse Hospital, Lyon University Hospital, Lyon, FRA; 3 Orthopaedic Department, General Hospital of Thessaloniki “Ippokrateio”, Thessaloniki, GRC; 4 2nd Orthopaedic Department, General Hospital of Thessaloniki "Papageorgiou", Thessaloniki, GRC

**Keywords:** blood loss, complications, total knee replacement, midvastus, medial parapatellar

## Abstract

Introduction

The aim of this study was to determine whether the midvastus (MV) approach in patients who underwent total knee replacement (TKR) results in differences regarding peri-operative parameters, such as surgical time, blood loss, transfusion need and complications, when compared with medial parapatellar approach (MP).

Methods

This was a prospective randomized comparative study of patients who received primary TKR. The first group consisted of patients where the MP approach was used and the second those where the MV approach was utilized. Patient’s age, body mass index (BMI), stage of osteoarthritis (OA), prosthesis design, duration of surgery, blood loss in the drainage, hemoglobin (Hb), and intra-operative complications were recorded.

Results

From December 2019 to June 2020 a total of 107 (22 males, 85 females) and 38 (seven males, 31 females) patients were operated on with the MP and MV approaches, respectively. The two groups did not differ in terms of age, BMI, gender and stage of OA, however, pre-operative haemoglobin (Hb) was higher in the MP group [mean 13,5 (1.3) versus 13.1 (0.73)]. There was no significant difference in Hb decline pre- and post-operatively and in drain volume between groups. The mean Hb drop was similar for the MP [-2.2 (1.08)] and MV [-2.52 (1.06)] groups, and even though the transfusion rates were lower for the MP group, it did not reach significance. The duration of surgery was significantly longer in the MV group, with a mean time of 95.6 (12.94) minutes versus 89.4 (14.28) in the MP group. Overall complications did not differ significantly among the two surgical approaches. Multivariate logistic regression demonstrated that pre-operative Hb [OR 2.6 95% CI (1.43, 4.75)] and approach [OR 4.15 95% CI (1.15, 14.98)] were significantly correlated with the need for transfusion when gender, BMI, redon drainage, prosthesis size and duration of surgery were considered together.

Conclusion

In our experience, total knee replacement performed with either the midvastus or medial parapatellar approach does not result in any advantage with regards to the intra-operative complications, drain blood volume or difference in Hb drop post-operative. However, the midvastus approach presents a longer operation time, with the risk of higher transfusion rates for the patients.

## Introduction

Knee osteoarthritis (KO) is a leading cause of chronic pain, disability and incremental healthcare costs among older adults. In recent years, overweight and obese people are up to four times more likely to develop KO due to an increase in mechanical load on the bearing joint [1]. Furthermore, the widespread media campaigns to encourage physical activity have been shown to elevate the risk of injury among the young population, increasing the risk of developing osteoarthritis [2]. In view of the above, there may be a greater need for joint replacement surgery even in patients aged over 45 years [1,2].

Knee replacement surgery has greatly evolved and surgeons employ various approaches to achieve the best outcome while minimizing damage to the extensor mechanism of the knee. The optimum approach is yet to be determined and no clear superiority is evident from studies comparing the traditional medial parapatellar (MP) with the midvastus (MV) approach [3,4]. Moreover, compared with the traditional MP, minimally invasive techniques, such as MV and subvastus (SV), which completely retain the extensor mechanism of the knee joint, showed favourable results in regards to the post-operative recovery of quadriceps function and pain [3-5], although there is no consensus regarding intra-operative parameters, like blood loss, tourniquet time, and peri-operative complications that favor one versus the other [5-8].

Thus, the aim of the present study was to investigate whether the MV approach is better than the MP in patients who underwent total knee replacement (TKR) regarding peri-operative parameters, such as surgical time, blood loss, transfusion need and complications.

## Materials and methods

This is a prospective randomized comparative study for the evaluation of peri-operative factors of total knee replacement between the MP and MV approaches, which was conducted at the 2nd Orthopaedic Department, General Hospital of Thessaloniki "Papageorgiou", Thessaloniki, Greece. From September 2019 to August 2020, a total of 145 patients were included in the present study. A voluntary informed written consent was obtained from all the patient or their legal guardian before or after enrolling in the study. The inclusion criterion was an indication for primary TKR (grade III and IV osteoarthritis according to the Kellgren-Lawrence classification system) due to primary and secondary osteoarthritis. Patients with inflammatory arthritis were excluded from the study.

Patients scheduled for primary TKR were divided into two groups according to the approach used. Patients were allocated by an independent secretary in the hospital’s administration office. All MV approaches were performed by the same surgeon, while TKR with the MP approach was done by two additional surgeons, both of whom had more than 10 years of experience. The first group consisted of patients in whom the MP approach was used and the second those with the MV approach. Patient’s age, body mass index (BMI), stage of osteoarthritis (OA) according to Kellgren-Lawrence classification, prosthesis design (CR, cruciate retaining or PS, posterior stabilizing), duration of surgery, blood loss in the drainage, hemoglobin (Hb), and intra- and post-operative complications were recorded. Hb was measured pre-operatively, as well as on the same afternoon of the operation and on the first post-operative day. Blood loss in the drain was recorded after removal 24 hours post-operatively, and whether patients required transfusion or not during hospitalization was also recorded. Peri-operative complications included partial patellar and popliteus tendon lacerations and excess bleeding after tourniquet release.

Surgical technique

A pneumatic tourniquet was used in all patients from the beginning of the procedure. In all patients, intravenous antibiotics with teicoplanin 400 mg bolus were administered 30 minutes prior to tourniquet inflation by the anesthetist and two additional doses post-operatively.

In the classic MP approach, a straight skin incision through the patellar midline down to the tibial tuberosity is performed, with the knee extended. The knee joint is accessed through dissection of the medial patellar retinaculum down to the tibial tuberosity and proximal to the medial third of the quadriceps tendon fibers, which are split longitudinally a few millimeters from its insertion to the patella. The patella is then everted and the knee flexed. Using TKR instrumentation, first, the distal femoral osteotomy is made followed by the tibial and then the anteroposterior osteotomies of the femur. An intramedullary guide was used for the femoral and extramedullary for the tibial axis evaluation in all patients. A drainage was used on all occasions and it was removed 24 hours post-operatively. Patients received opioid and paracetamol analgesia post-operatively and low molecular weight heparin as antithrombotic prophylaxis for one month. On the first post-operative day, a well-supervised rehabilitation program was begun, consisting of isometric quadriceps and active knee range of motion exercises, in order to improve the overall functional outcomes of the patients.

In the MV approach, a longitudinal skin incision is made along the medial third of the patella extending 2 cm proximal to the proximal pole of the patella down to the tibial tuberosity, with the knee flexed. The joint is accessed by dissection of the medial patellar retinaculum extending proximal through an oblique incision in line with the fibers of the vastus medialis oblique 2-3 cm long. The patella is displaced laterally but not everted. The rest of the stages using TKR instrumentation were the same as previously mentioned. 

Statistical analysis

Statistical analysis was performed using the GNU PSPP 1.4.1-2 software (Free Software Foundation, Boston, USA). Demographic characteristics are presented as means, standard deviations and percentages. For comparison of categorical variables Pearson’s Chi-square test was used, and for continuous variables paired and unpaired samples t-test, because data were not skewed. Multiple logistic regression was utilized to find factors associated with transfusion when all variables were considered together. Statistical significance was set at p < 0.05.

This study received Institutional Board Approval (Reference Number: 75-334/17.09.2020).

## Results

A total of 145 patients underwent unilateral TKR in a single tertiary center, among which 107 patients were in the MP group (22 male and 85 female) and 38 patients were in the MV group (seven male and 31 female) with mean age of 71.3 (6.1) and 73.1 (5.06) years, respectively. There were no significant differences between groups regarding gender, age, BMI, OA stage and prosthesis design (p>0.05), but there was a highly significant difference in pre-operative Hb with the MV group being lower, 13.1 (0.73) (p=0.04) (Table 1). In most cases, the CR prosthesis design was used in both groups (90 prostheses in the MP group and 31 prostheses in the MV group).

**Table 1:** Demographic characteristics of patients undergoing total knee replacement. Abbreviations: MP, medial parapatellar approach; MV, midvastus approach; M, male; F, female; BMI, body mass index; OA, osteoarthritis; CR, cruciate retaining; PS, posterior stabilizing; n, number; Hb, hemoglobin Data are expressed as mean ± standard deviation (S.D.); ^†^ Data is expressed as median (interquartile range); ^‡^ Kellgren-Lawrence Classification for OA

Characteristics	MP (n = 107)	MV (n = 38)	p-value
Gender, M/F (%)	21.3/78.7	18.4/81.6	0.11
Age (years)	71.36 (6.14)	73.16 (5.06)	0.09
BMI^†^ (kg/m^2^)	31.6 (6.5)	32 (8.6)	0.13
OA stage^‡^, 1/2/3/4 (%)	0.9/1.9/34.6/62.6	-/-/36.8/63.2	0.77
Prosthesis design, CR/PS (%)	84.1/15.9	81.6/18.4	0.71
Pre-operative Hb (g/dl)	13.54 (1.33)	13.18 (0.73)	0.04

There was no significant difference in Hb decline pre- and post-operatively in drain volume or transfusion rates between groups. The mean Hb drop was similar for the MP and MV groups, and even though transfusion rates were lower for group MP, it did not reach significance (Table 2). The latter probably reflects the effect of higher pre-operative Hb in the MP group compared to the MV group. There were five partial patella tendon lacerations and three partial popliteus lacerations in the MP group versus four partial patella tendon lacerations in the MV group. Overall peri-operative complication rates did not reach statistical significance. The duration of surgery was significantly longer in the MV group, with a mean time of 95.6 minutes versus 89.4 minutes in the MP group (Table 2).

**Table 2:** Comparisons of peri-operative parameters between the two approaches Abbreviations: MP, medial parapatellar approach; MV, midvastus approach; Hb, hemoglobin Data is expressed as mean ± standard deviation (S.D.); ^†^ Data is expressed as median (interquartile range); ^‡^ Pearson’s Chi-square = 2.86 (degrees of freedom 1); ^^^ Pearson’s Chi square = 0.76 (degrees of freedom 1)

Peri-operative parameters	MP (n = 107)	MV (n = 38)	p-value
Duration of surgery (min)	89.4 (14.3)	95.6 (12.9)	0.02
Drain volume^†^ (ml)	380 (280)	350 (237.5)	0.63
Hb difference^†^ (g/dl)	2.2 (1.1)	2.52 (1.1)	0.13
Transfusion (%)	10.3	21.1	0.09^‡^
Complications (%)	7.6	3.5	0.59^^^

**Figure 1:**
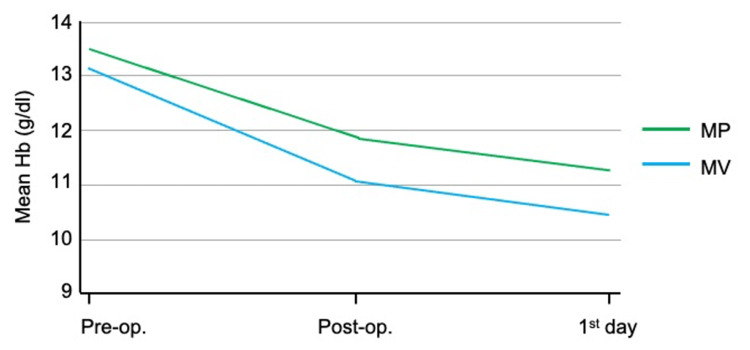
Post-operative changes in hemoglobin (Hb) among the two surgical approaches.

In terms of blood loss, both groups demonstrated the same tendency in Hb gradual drop, as measured in the immediate post-operative period and the first post-operative day (Figure 1). Multiple logistic regression was used to assess transfusion requirements, taking into consideration gender, BMI, pre-operative Hb, redon drainage, approach, prosthesis size and duration of surgery. Significant factors were pre-operative Hb and surgical approach. Specifically, for every 1 unit of rise in Hb, the probability of a patient not needing transfusion was 2.6 times greater, while when the medial parapatellar approach was used there was four times more probability that the patient would not be transfused but with a very wide confidence interval, when all the aforementioned factors were considered together.

## Discussion

The accumulated experience of orthopedic surgeons regarding TKR over the years and the need to reduce soft tissue damage, especially in the extensor mechanism of the knee, has led to the description of various new approaches, apart from the traditional medial parapatellar [9]. Among the latter is the MV, where the vastus medialis is split along its fibers, providing good exposure of the joint and preserving the quadriceps tendon [10].

Our study did not demonstrate significant differences in intra-operative complications between patients undergoing MP and those undergoing the MV approach, with the exception of an increased proportion of patients in the MV approach who had a higher rate of blood transfusions and more surgical time. It has been suggested that blood loss during TKR is putting the patients at risk for an allogenic blood transfusion [11]. Considering that, peri-operative blood management strategies have been established in order to minimize blood loss and the additional need for allogenic blood transfusion [11,12]. Previously published studies have shown possible predisposing factors [11-13]. Among them, pre-operative anemia (or Hb < 12 g/dl), non-obese population (BMI < 30), drainage volume (> 500 ml) and total operative time (> 2 hours) are significant factors of peri-operative blood transfusion [11,13-15].

Our results demonstrated that pre-operative level of hemoglobin is a significant risk factor for allogenic blood transfusion, indicating that for every 1 unit of rise in Hb, the probability of a patient not needing transfusion was 2.6 times greater. This finding was consistent with other previous comparative studies. Cao, et al. found that lower pre-operative hemoglobin (12.1 g/dl) and more intra-operative blood loss (130.9 ml) were independent risk factors for post-operative anemia [16]. In a large retrospective study with 2284 patients undergoing TKR, the authors reported that pre-operative anemia increased the rate of transfusion by 6.38 and 6.27 times in males and females, respectively [17]. In view of the above, correction of pre-operative low levels of hemoglobin by pre-operative blood donation-induced stimulation of erythropoiesis before elective surgeries, such as TKR, or other measures should always be considered [11,14].

Findings from our study indicated that in the patients in whom the medial parapatellar approach was used, there was four times more probability that the patient would not be transfused. Feczko, et al. reported significantly higher blood loss in the midvastus group as compared to the parapatellar group, without indicating the need for blood transfusion [18]. The authors considered this as a result of the elongated surgical time. Contrary to our study, Chaiyakit, et al. in a recent retrospective study compared the amount of blood transfusion after TKR to the surgical technique [19]. They found that computer-assisted and minimally invasive surgical techniques had decreased the risk of blood transfusion. This fact is based on the theory that small incisions cause less muscle and soft-tissue damage leading to less blood loss. On the other hand, computer-assisted and minimally invasive surgery in TKR tend to increase the overall operation period and the possibility of more blood loss [7,20].

TKR is a common and safe procedure that relieves the symptoms of arthritis and improves the quality of life [12]. Nevertheless, like any major surgical procedure, TKR is not without complications, with rates reaching up to 20% for older patients [21]. The most often referenced complications in the literature are cardiovascular, respiratory and thromboembolic episodes [12,13]. Interestingly, several studies have also evaluated intra-operative iatrogenic complications during revision TKR, but there is limited data on intra-operative iatrogenic complications during primary TKR [22]. The rates of complication vary from 0.2% to 4.4%, while it depends on patient factors, the type of implants used and the surgical technique [12,22-25]. Among them, the most frequently reported complications in the literature are iatrogenic laceration of the popliteus tendon, fractures and vascular injuries [23,24,26]. However, the incidence of iatrogenic complications in our study was quite high, with the majority occurring in posterior stabilized TKR and with the midvastus approach, suggesting the need for further caution with the femoral/tibial cut technique and the eversion of the patella. As a result, it is of great importance for clinicians to be aware of the risk factors that are associated with these complications to minimize them.

The present study has some limitations. First, the relatively small sample size of the total number of TKRs limits the multivariable analyses and the ability to compare the characteristics between the two main surgical approaches. In particular, the data were not extracted from multiple centers but from a single tertiary hospital, and there were more patients in the MP group than the MV. A second limitation is that surgeries were not performed by the same surgeon; there were multiple surgeons involved, thus introducing performance bias to the study, and there was no randomization process. Thirdly, even though we checked for confounding factors using logistic regression analysis, selection bias cannot be removed because we did not perform propensity score analysis to control for unmeasured confounders. Despite these limitations, the current prospective study followed appropriate methodology and provided useful information about the peri-operative complications in people undergoing primary TKR.

## Conclusions

In the current study, we did not observe any benefit of the MP and MV approaches to the primary TKR with regard to the intra-operative complications, blood loss in the drainage or difference in Hb decline post-operative. Although, the MP approach showed less surgical time and less need for transfusion when compared to the MV approach.
